# Radial glia fibers translate *Fgf8* morphogenetic signals to generate a thalamic nuclear complex protomap in the mantle layer

**DOI:** 10.1007/s00429-018-1794-y

**Published:** 2018-11-23

**Authors:** Arancha Botella-López, Raquel Garcia-Lopez, Ana Pombero, Salvador Martinez

**Affiliations:** 1 Instituto de Neurociencias UMH-CSIC, Alicante, Spain; 2 IMIB-Arrixaca, ISCIII and University of Murcia, Murcia, Spain; 3 CIBER de Salud Mental CIBERSAM ISCIII, Murcia, Spain

**Keywords:** *Fgf8*, Thalamus, Positional information, Diencephalon development, Brain regionalization, Thalamocortical projection

## Abstract

Thalamic neurons are distributed between different nuclear groups of the thalamic multinuclear complex; they develop topologically ordered specific projections that convey information on voluntary motor programs and sensory modalities to functional areas in the cerebral cortex. Since thalamic neurons present a homogeneous morphology, their functional specificity is derived from their afferent and efferent connectivity. Adequate development of thalamic afferent and efferent connections depends on guide signals that bind receptors in nuclear neuropils and axonal growth cones, respectively. These are finally regulated by regionalization processes in the thalamic neurons, codifying topological information. In this work, we studied the role of *Fgf8* morphogenetic signaling in establishing the molecular thalamic protomap, which was revealed by *Igsf21, Pde10a* and *Btbd3* gene expression in the thalamic mantle layer. *Fgf8* signaling activity was evidenced by pERK expression in radial glia cells and fibers, which may represent a scaffold that translates neuroepithelial positional information to the mantle layer. In this work, we describe the fact that Fgf8-hypomorphic mice did not express pERK in radial glia cells and fibers and presented disorganized thalamic regionalization, increasing neuronal death in the ventro-lateral thalamus and strong disruption of thalamocortical projections. In conclusion, *Fgf8* encodes the positional information required for thalamic nuclear regionalization and the development of thalamocortical projections.

**Electronic supplementary material:**

The online version of this article (10.1007/s00429-018-1794-y) contains supplementary material, which is available to authorized users.

## Introduction

Thalamic projection to specific areas of the cortex (Cx) is fundamental in the development of conscious sensory and motor functions, and thalamic neurons projecting to the Cx present homogeneous morphology (Jones 2007; Clascá et al. 2012). Nevertheless, target specificity in the thalamocortical projection (TC) constitutes one of the most prominent high-level processes in the developing mammalian brain (Jones 2001; Clascá et al. 2009; Sherman and Guillery 2011). The organized pattern of TC is a consequence of elaborate mechanisms of positional information in thalamic neurons and axonal navigation (Garel and López-Bendito 2014). The functional complexity of this system requires positional information, which is represented in the germinative epithelium of the diencephalon (Di) and Cx as molecular maps specifying neural identities (Rakic 1988; O’Leary et al. 2007; Nakagawa and Shimogori 2012; Martinez-Ferre and Martinez 2012; Elsen et al. 2013).

The expressions of *Netrin1* (*Ntn1*), *Slit 1, Robo, Sema3A* and *EphrinA5* in the ventral telencephalon (VTel) act as molecular signals for thalamic axon navigation, to innervate the Cx (Andrews 2006; Bagri et al. 2002; Bielle et al. 2011; Bonnin et al. 2007; Braisted et al. 2000; López-Bendito et al. 2006; Serafini et al. 1996). Axons grow from the thalamus (Th) to prethalamus (PTh), entering the internal capsule between embryonic days 12–15 (E12 to E15), reaching the cortical subplate (SP) at E14-16, and invading Cx by E18 (Ghosh et al. 1990; Squarzoni 2015).

During early embryogenesis (E9-E11), diencephalic neuroepithelial regions are specified by the expression of genes coding for morphogenetic signals and molecular regulators of cellular identities (Puelles et al. 1987; Puelles and Rubenstein 1993; Stoykova and Gruss 1994; Redies 1995; Redies et al. 2000; Kataoka and Shimogori 2008; Suzuki-Hirano et al. 2010; Nakagawa and Shimogori 2012; Navarro-Garberi et al. 2016). Therefore, neuroepithelial regionalization consists in a primordial bidimensional (2D) molecular map that defines antero-posterior (AP) and ventro-dorsal (VD) positional information. Subsequently, postmitotic neurons migrate into the thalamic mantle layer (TML) to either become grouped into compact masses (thalamic nuclei) or remain disaggregated inside crossing axonal tracts (reticular thalamic areas). According to their position in the TML, these neurons will express specific genetic patterns and follow different programs of structural and functional maturation. However, little is known about how regional neuroepithelial information is translated into the TML. To unravel the process of TML regionalization; that is, how the neuroepithelial map is translated to the TML and its role in the final neuronal fate, we analyzed the expression of three genes: *Mus musculus immunoglobulin superfamily, member 21* (*Igsf21*); *Phosphodiesterase 10A* (*Pde10a*) and *BTB* (*POZ*) *domain containing 3* (*Btbd3*). These genes exhibit a complementary expression pattern in the TML at E13.5 and E14.5, when the TML is still a structurally homogeneous mass of neurons, and TC pioneer axons are reaching the Cx. Next, we explored whether TML regionalization is regulated by signals that specify the neuroepithelial map. To this end, since the *Fibroblast growth factor 8* (*Fgf8*) gene is expressed in the dorsal diencephalon (Supplementary Fig. S1) and the *Fgf8* signal is involved in specifying thalamic progenitors (Kataoka and Shimogori 2008; Martinez-Ferre and Martinez 2009), the thalamic regionalization and TC projection were studied in *Fgf8* hypomorphic mice (Fgf8^null/neo^), which express very low levels of *Fgf8* in the Di (Meyers et al. 1998; Martinez-Ferre and Martinez 2009). Previously, we studied the role of *Fgf8* in early diencephalic development, also using *Fgf8* hypomorphic mice, showing that the progressive reduction of *Fgf8* expression in the dorsal diencephalon results in increasing alterations of cellular proliferation and neuronal migration. Although *Fgf8* was involved in specifying epithalamic and thalamic growth, no effect was observed on the formation of the *zona limitans* and early diencephalic regionalization (Martinez-Ferre and Martinez 2009). Therefore, we decided to analyze the potential role of *Fgf8* in later-stage TML regionalization.

Moreover, although previous studies have related *Fgf8* to axonal guidance (Garel et al. 2003; Yamauchi et al. 2009; Atkinson-Leadbeater et al. 2010), we also wanted to study the role of this morphogen in TC development.

*Fgf8* signaling activity in TML cells was studied through the activation of *pERK* expression (Echevarria et al. 2005), which was detected in diencephalic radial glia cells and fibers. We showed that *pERK* expression in the radial glia controls the proliferation of thalamic progenitors, as well as the survival and regionalization of neurons in the TML. We also evidenced a prominent role of the *Fgf8* signal in thalamic axon navigation to the Cx. These findings demonstrate that radial glia are a fundamental scaffold for translating 2D maps of ventricular diencephalic specification to the mantle layer, where a 3D thalamic protomap is established, regulating neuronal survival and thalamic nuclear specialization.

## Materials and methods

### Mouse lines and genotyping

All animal experiments were performed in compliance with Spanish (RD 223/1998) and European Union laws on the protection of animals used in experimentation (Council Directive 86/609/EEC) and approved by the UMH-CEIE committee.

Fgf8^neo/+^ and Fgf8^null/+^ mice were bred to produce Fgf8^null/neo^ mutants (C57BL/6 genetic background). PCR genotyping was performed as described by (Chi et al. 2003). The morning the vaginal plug was detected was considered embryonic day 0.5 (E0.5). Heterozygous Fgf8^neo/+^, Fgf8^null/+^ embryos did not present any different structural phenotype and were used together with Fgf8^+/+^ embryos as controls.

The mouse embryos were fixed overnight by immersion in 4% paraformaldehyde in phosphate-buffered saline solution (PBS; 0.1M, pH 7.4) at 4 °C.

### In situ hybridization

The mouse brains were paraffin embedded (Gemcut, Spiele no. 24364-1) and sectioned in 7 µm-thick sagittal and transverse sections. The sections were washed with phosphate-buffered saline (PBS; pH 7.4, 1X)-0.1% Tween-20 (Sigma Aldrich, Steinhem, Germany) and incubated in hybridization solution: deionized formamide 50%, standard SALT buffer 1X pH 7.5, Dextran sulfate 10X (Sigma-Aldrich), Denhart 1X (Sigma-Aldrich), yeast tRNA 2 mg/ml (Sigma-Aldrich) and water free of RNAase and DNAase (Sigma-Aldrich), for 1 h at 67 °C. Thereafter, the sections were hybridized overnight at 67 °C in hybridization solution containing 4 µl/ml riboprobe. Next, they were incubated overnight at 4 °C with alkaline phosphatase-coupled anti-digoxigenin antiserum (1:3500; Roche Diagnostics, Mannheim, Germany); followed by NBT/BCIP (nitroblue tetrazolium/5-bromo-4-chloro-3-indolyl phosphate) solution as a chromogenic substrate to obtain blue labelling (Boehringer, Mannheim, Germany). The sections were dehydrated and covered with Eukitt^®^. All images were taken using a Leica stereoscope (Leica MZ16FA) and digital cameras (Leica DC500, DC250). The contrast and brightness of the photomicrographs were adjusted in Adobe PhotoShop, Macintosh or PC version, CS3 (Adobe Systems, San Jose, CA). Digoxigenin-11-UTP labelled RNA probes were prepared from full-length cDNA clones provided by Source BioScience imaGenes: Igsf21 (NM_198610.2) 32-1990 pb, Pde10a (NM_011866.2) 402-3324 pb; Btbd3 (NM_145534.2) 30-2439 pb. The *Ntn1* probe was provided by O. Reiner, and *Slit1* and *Slit2* probes by O. Marín.

### Immunohistochemistry

Paraffin sections were treated with 0.3% hydrogen peroxide in PBS + 0.3% Triton (PBT) for 15 min to inactivate endogenous peroxidase activity, blocked in PBT and 3% BSA and incubated with anti-DCC antibody (1:100; SantaCruz #sc-6535). Then they were incubated with secondary biotinylated antiserum for 2 h at room temperature (RT; Vector, Burlingame, CA, USA), washed with PBT and incubated in avidin–biotin complex (ABC kit; Vector; 0.003% dilution) for 1 h at RT. The immunolabeling was revealed by 0.05% diaminobenzidine (DAB; Vector Laboratories SK-4100) in 0.05 M Tris buffer (pH 7.6), containing 0.03% H_2_O_2_. For anti-dpERK immunohistochemistry (Rabbit anti-dpERK, 1∶250; Cell Signaling Technology #9101), the brains were embedded in 4% agarose in PBS, and 70 µm sections were cut in coronal planes using a Leica vibratome (VT1000S). For these sections, the immunolabelling was revealed with 0.05% DAB, 0.025% ammonium nickel sulfate hexahydrate and 0.03% H_2_O_2_ in PBS.

Fluorescent immunohistochemistry: paraffin sections were incubated overnight with rabbit anti-phospho histone 3 (1:500; Upstate #06-570), rabbit anti-Caspase 3 (1:250; Cell Signaling #9661-s), rabbit anti-dpERK (1∶250; Cell Signaling Technology #9101) and mouse anti-GFAP (1:1000; Millipore #MAB360). After this, sections were incubated with anti-rabbit Alexa Fluor 488 (Molecular Probes #21206) and anti-mouse biotinylated antibody (1:200;Vector #BA-9200), followed by Cy3-streptavidin (1:500; Amersham PA#43001) and then counterstained with DAPI (4′, 6-Diamidino-2-Phenylindole, dihydrochloride; Sigma) a fluorescent nuclear dye, diluted in PBS at 0.001% and incubated for 10 min at RT.

### Cell proliferation and apoptosis analysis

In order to assess possible alterations in proliferation or cell death in Fgf8^null/neo^ mutants paraffin sections were immunostained with anti-phospho histone 3 and anti-Caspase 3, as described above. Then, the number of positive cells for the corresponding antibody was quantified by counting positive cells per unit area (mitotic index). This unit area was a box of 10500 µm^2^ placed on the neuroepithelium from the epithalamus to the thalamus, following a previously described protocol (Martinez-Ferre and Martinez 2009). Three consecutive sections were counted per animal and three animals were used for each stage and genotype.

### Organotypic cultures

E13.5 organotypic cultures of embryonic mouse brains (Echevarría et al. 2001): the neural tube, like an “open book”, was transferred to sterile Petri dishes and placed on floating polycarbonate membrane 8 µm pore size filters (Nunc) with 10% fetal bovine serum in DMEM culture medium (Gibco-Life Technologies). Glutamax (2 mM; Gibco-Life Technologies) and Penicillin–Streptomycin (100U/ ml–100 µg/ml; Gibco-Life Technologies) were added to the culture medium. The organotypic cultures were incubated for 1 h in a sterile incubator (37 °C, 5% CO_2_), after which the medium was changed to Neurobasal/B-27 (Gibco BRL, Life Technologies Inc, Gaithersburg, MD).

Beads were implanted in the dorsal Di at E13.5 as described previously (Echevarría et al. 2001; Martinez-Ferre and Martinez 2009). Affi-Gel Blue Gel beads (Bio-Rad) were rinsed in PBS and then soaked overnight at 4 °C in a solution of 25 µg/ml Dkk-1 protein in PBS/0.1% BSA (Sigma), or with the FGF8 inhibitor SU5402 40 µM (Calbiochem, La Jolla, CA, USA) in dimethyl sulfoxide (DMSO; Sigma), respectively. The beads were then rinsed several times in 1 mg/ml BSA solution or DMSO and implanted into the explants. For the control experiments, beads were soaked in PBS/0.1% BSA in the same manner. The embryos were fixed in 4% PFA at 4 °C, overnight, 2 days after implantation.

In some cases, the organotypic cultures were paraffin embedded, cut and processed immunohistochemically using Rabbit anti-Caspase-3 (1:250; Cell Signaling Technology #9661).

### Axonal Tracing

For axonal tracing, small DiI crystals (1,1′-dioctadecyl 3,3,3′,3′-tetramethylindocarbocyanine perchlorate; Molecular Probes) were inserted into the Th of hemi-dissected brains. These embryonic brains or cultured slices were incubated for 7 days, or 2 days, respectively, in 4% PFA at 37 °C. The brains were cut using a Leica vibratome (VT1000S) into 80–100 µm sections and counterstained with DAPI.

## Results

### Disorganization of thalamic regionalization in Fgf8^null/neo^ hypomorphic mice

**Fig. 1:**
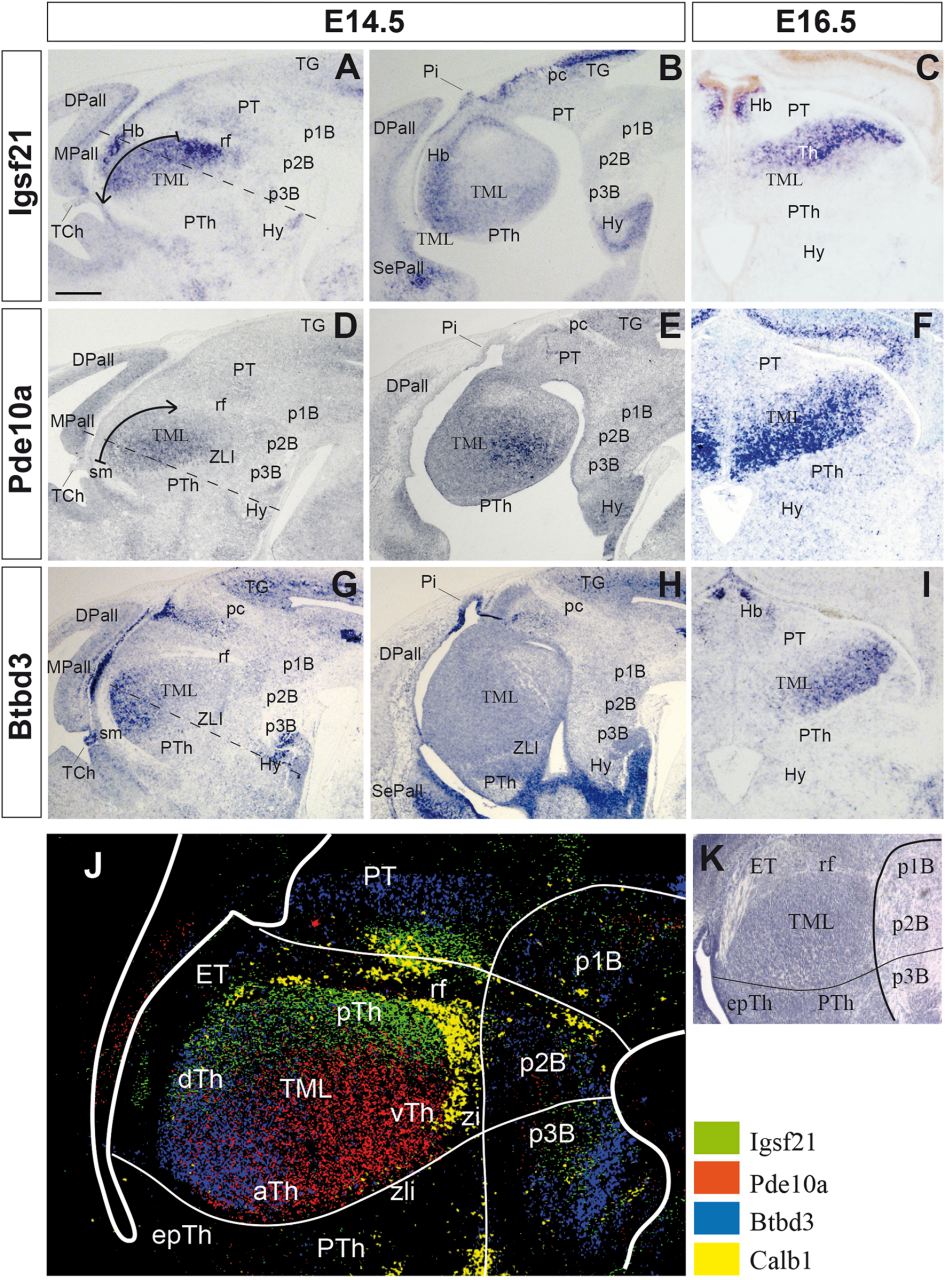
*Igsf21, Pde10a* and *Btbd3* expression in the developing thalamus of a mouse. *Igsf21, Pde10a* and *Btbd3* expression in E14.5 and E16.5 embryos, sagittal (lateral to medial, **a, b; d, e; g, h**) and transversal (**c, f, i**) sections. **a**–**c** Thalamic *Igsf21* expression is detected as a posteriorly to anteriorly decreasing gradient (arrow; **a**). **d**–**f***Pde10a* is mainly detected in the anterior Th, with a rostro-caudal decreasing gradient (arrow; **d**). **g**–**i***Btbd3* is expressed in the most dorsal region, showing a dorso-ventral gradient. **j** Complementary expression of these genes in the thalamic mantle layer, shown in a composite picture using equivalent sagittal section planes aligned by overlapping three stable anatomical references (rf, mamillary bodies, ETh-telencephalic flexure). Color-coded overlays represent different gene expression patterns (see insert). **k** Nissl’s staining of E14.5 Th, showing the homogeneous distribution of neurons in the mantle layer. Dashed lines in **a, d** and **g** correspond to the plane of the transversal sections (**c, f, i**). Scale bar 200 µm

**Fig. 2:**
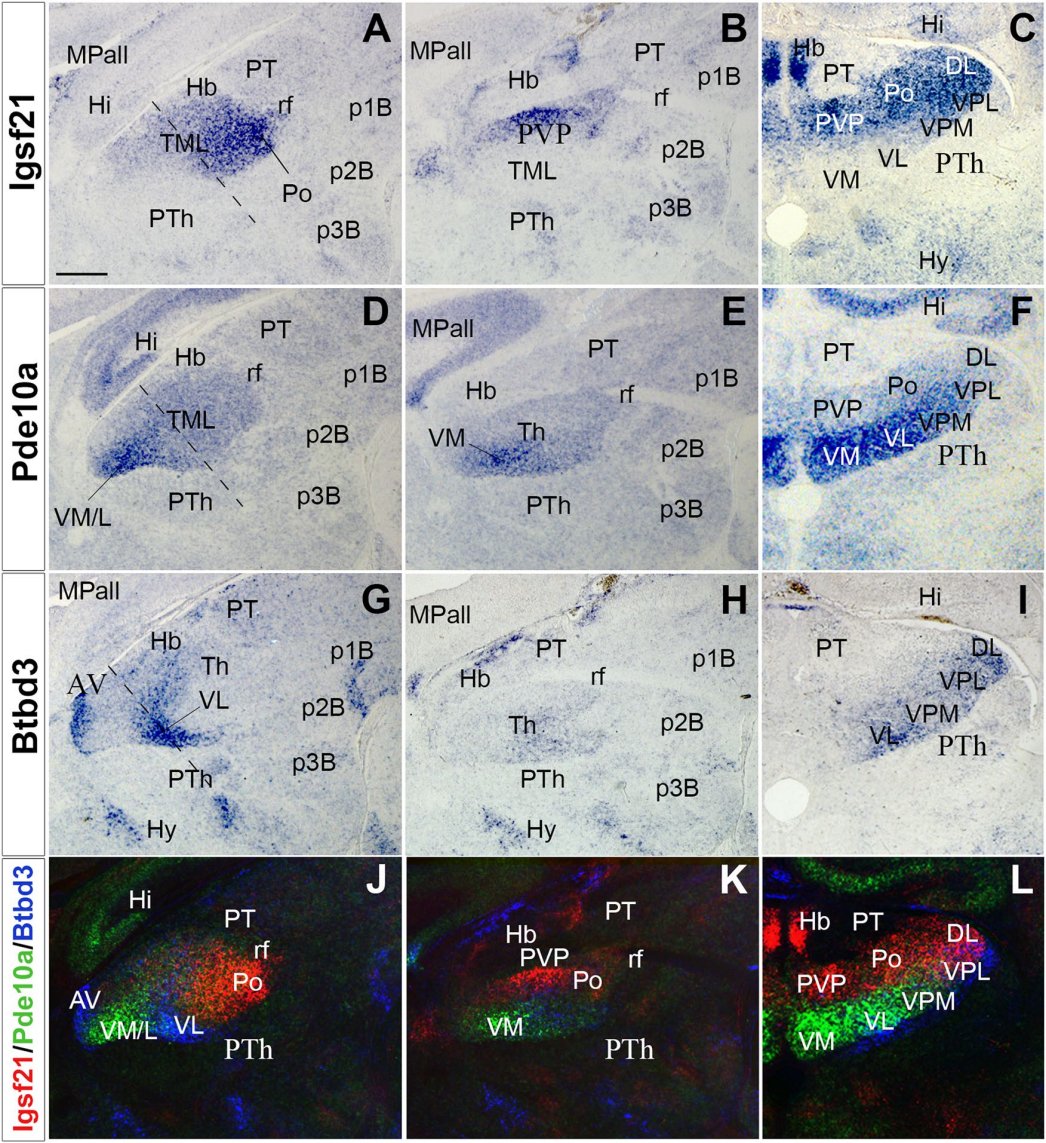
*Igsf21, Pde10a* and *Btbd3* expression in perinatal stages. *Igsf21, Pde10a* and *Btbd3* expression in E17.5 embryos, sagittal (lateral to medial, **a, b; d, e; g, h**) and transversal (**c, f, i**) sections. Color-coded overlays of hybridizations for *Igsf21* (red), *Pde10a* (green) and *Btbd3* (blue) genes (**j**–**l**). **a**–**c***Igsf21* is strongly expressed in Po and DL nuclei. **d**–**f***Pde10a* is strongly labelled in the VM, VL and VPM. **g**–**i***Btbd3* displays a weak signal in VL, VPM, VPL and DL nuclei. **j**–**l** pseudo-colored superposition of adjacent sections depicting the complementary expression of the three genes. The molecular boundary between dorsal and ventral nuclei is clearly detectable, mainly between *Igsf21* and *Pde10a* expression domains. Dashed lines in **a, d** and **g** correspond to the plane of the transversal sections (**c, f, i**). Scale bar: 200 µm

At E13.5-14.5, the TML seems to be a structurally homogeneous mass of neurons (Nakagawa and O’Leary 2003) (Fig. 1; Fig. S2). To explore whether TML is already molecularly patterned at these stages, transcription databases were assessed to identify genes with heterogeneous expression in the TML (http://www.eurexpress.org and http://www.brain-map.org). As a result, 90 genes were identified with expression in the TML at E13.5-14.5, but only 12 genes showed clear regional expression: *BC055811, BC062109, Btbd3, EG628779, Elmo1, Igsf2, Pde10a, Rapgef3, Slc6a7, Slitrk6, Srgap2* and *Trim9*. From these, we selected three showing clear heterogeneous complementary expression in the TML: *Igsf21, Pde10a* and *Btbd3* (Figs. 1, 2). Thus, their expression pattern was studied in the TML from E14.5 to E17.5. First, a decreasing caudal-to-rostral gradient of *Igsf21* expression was observed at E14.5 (Fig. 1a, b). Conversely, *Pde10a* transcripts showed a decreasing rostral-to-caudal gradient (Fig. 1d, e), whereas *Btbd3* displayed a decreasing dorsal-to-ventral gradient in the TML (Fig. 1g, h). Second, at E16.5, *Igsf21* expression was detected in the caudal and dorsal Th (Fig. 1c). *Pde10a*-expressing cells were mainly mapped in the anterior and ventral TML, with stronger medial than lateral expression (Fig. 1f), while *Btbd3* was restricted to the most ventrolateral region (Fig. 1i). Finally, at E17.5, when nuclear segregation was clear in the TML, *Igsf21* was expressed in the posterior and dorsal thalamic nuclei (Fig. 2a–c), whereas *Pde10a* showed decreasing medial-to-lateral expression in the ventral thalamic nuclei (Fig. 2d–f). Furthermore, a *Btbd3* signal (Fig. 2g–i) was detected in the anterior nucleus (Fig. 2g), showing a decreasing lateral-to-medial gradient in the ventrolateral nuclei (VL; Fig. 2g, i). The complementarity between these expressions in the TML was clear when adjacent sections were combined, matching at least three stable anatomical references to superpose three consecutive sections, both in sagittal and transverse sections (Figs. 1j, 2j, l). These compositions were generated by combining images from consecutive series from the same animal to establish accurate overlapping. Dorsal and posterior nuclei expressed *Igsf21*, ventromedial nuclei expressed *Pde10a*, and VL expressed *Btbd3*. A sharp molecular boundary was detected along the radial extension of the TML, separating the dorsal and ventral nuclei, where the thalamic internal medullary lamina (iml) would develop (Fig. 2l).

**Fig. 3:**
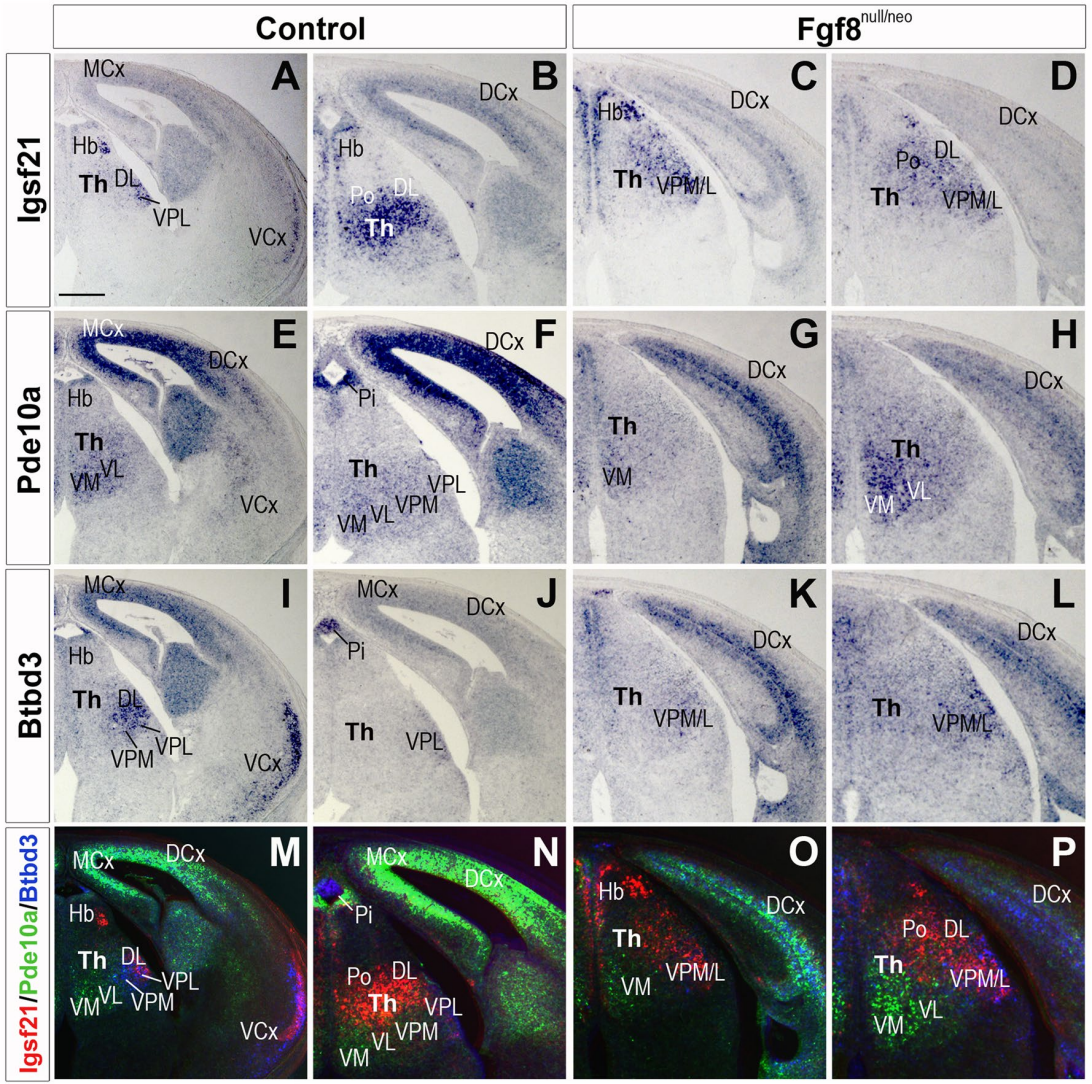
*Igsf21, Pde10a* and *Btbd3* expression in control and Fgf8^null/neo^ mice at E15.5. **a**–**d***Igsf21* expression, **e**–**h***Pde10a* expression, **i, l***Btbd3* expression and **m**–**p** overlay of the three gene expressions in control and Fgf8^null/neo^ embryos, transverse sections. *Igsf21* is detected mainly in the thalamic DL and Po nuclei in the control mice (**a, b**). In Fgf8^null/neo^ mice, *Igsf21* is downregulated in DL/Po and ectopically detected in abundant cells of VPM and VPL nuclei (**c, d**). *Pde10a* is expressed in the ventral nuclei, VM, VL, VPM and VPL, in controls (**e, f**) while it is upregulated and in the mutant VM nucleus, which appears extended dorsally (**g, h**). *Btbd3* is weakly detected in VPM and VPL nuclei in Fgf8^null/neo^ (**k, l**) compared to controls (**i, j**). Color-coded overlays depict the complementary expression gradient of the three genes in the control (**m, n**), and diffuse labelling in the mutant mouse (**o, p**). The molecular boundary between dorsal and ventral nuclei appears disrupted by intermingled cell expression of dorsal and ventral markers. Scale bar 200 µm

**Fig. 4:**
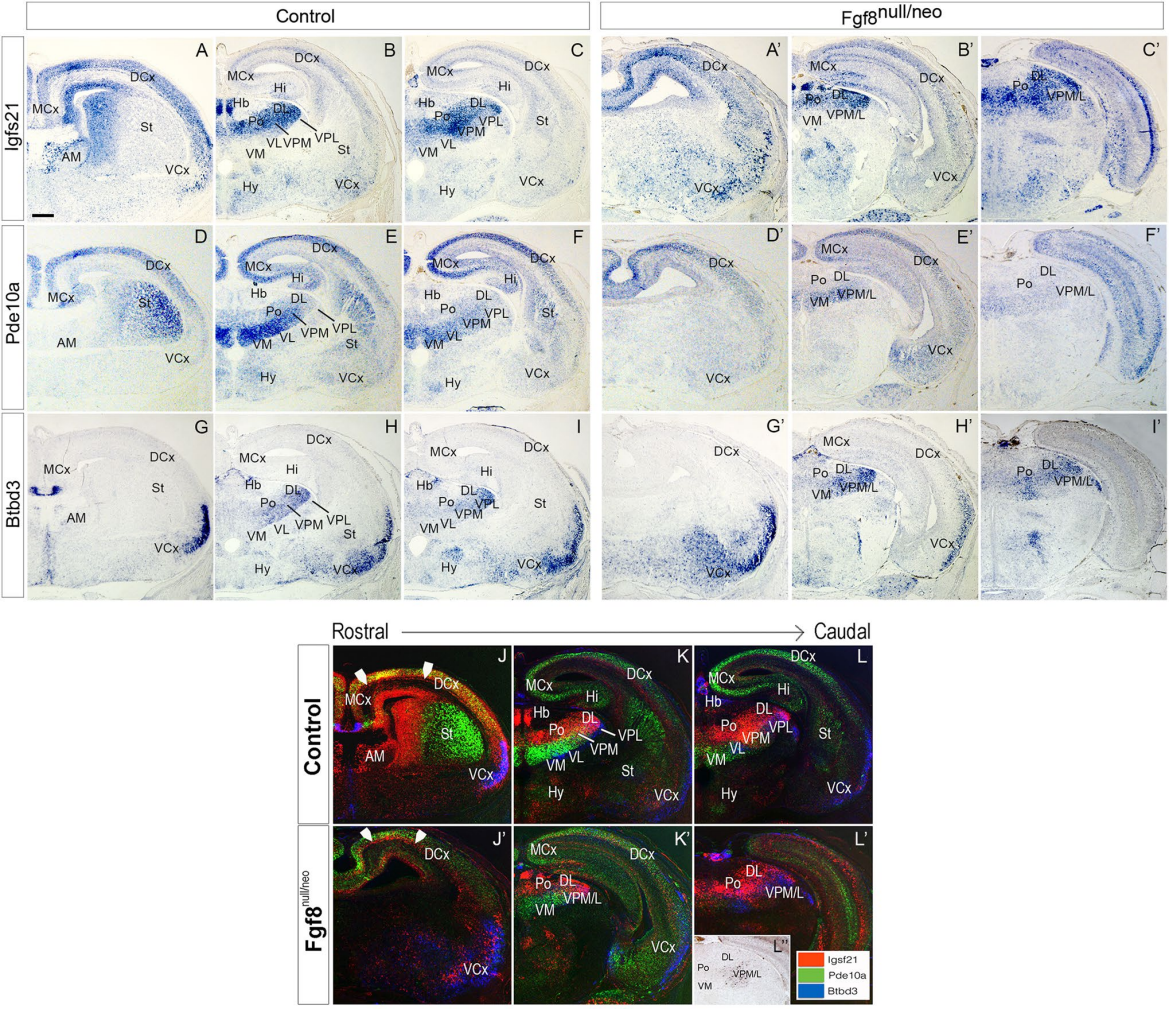
*Igsf21, Pde10a* and *Btbd3* expression in the Cx of a Fgf8^null/neo^ mouse in perinatal stages (E17.5). *In situ* mRNA hybridization in transversal brain sections of *Igsf21, Pde10a* and *Btbd3* genes in control (**a**–**i**) and in Fgf8^null/neo^ (**a**′–**i**′) mice. Images are adjacent sections, ordered from rostral to caudal, obtained from the same animal to match the different areas accurately (**j**–**l**′). Color-coded overlays of hybridizations for *Igsf21* (red), *Pde10a* (green) and *Btbd3* (blue) genes in E17.5 transversal sections of control (**j**–**l**) and Fgf8^null/neo^ (**j**′–**l**′) embryos, from rostral to caudal (**j** to **l** and **j**′ to **l**′, respectively). Arrowheads indicate the clear subplate cell layer (SP) in the wild type (**a**) that appears disorganized in the Fgf8^null/neo^ mouse. The overlap of the three genes confirms the complementary expression pattern in this region in the control, but not Fgf8^null/neo^ mice, where the anterior nuclear complex is not apparent and there is a reduction of ventral-lateral nuclei, which are invaded by the dorso-lin ateral marker gene. The boundary between dorsal and ventral nuclei is disrupted by bidirectional cellular dispersion across its domain. **l**″ is a caudal transversal section from an Fgf8^null/neo^ mutant mouse, immunostained against caspase 3, where VPM/L is clearly affected. Scale bar: 200 µm

To determine the role of *Fgf8* in TML regionalization, we studied the expression patterns of *Igsf21, Pde10a* and *Btbd3* when *Fgf8* transcription was strongly reduced. Since previous data indicated that changes in TML regionalization occurs after E14.5 (Martinez-Ferre and Martinez 2009), we decided to start our analysis at E15.5 stage. Thus, E15.5 Fgf8^null/neo^ embryos were analyzed together with littermate controls (Fig. 3). In transversal sections of Fgf8^null/neo^ embryos (*n* = 4), *Igsf21* transcripts were reduced in Po and DL nuclei (Fig. 3a–d) compared to controls (*n* = 4). Moreover, upregulation of this gene was detected in scattered cells of the lateral ventral nuclei, VPM/L (Fig. 3c, d). The *Pde10a* gene was upregulated in medio-ventral nuclei, which extended dorsally in Fgf8^null/neo^ mice (*n* = 4) and downregulated in the VPM/L (*n* = 4; Fig. 3e–h). The expression of *Btbd3* was predominantly observed in the VPM/L and DL nuclei in control mice (Fig. 3i, j), being expressed more weakly in the mutant Th (Fig. 3k, l). At E17.5, the TML of hypomorphic mice (*n* = 4) was clearly smaller, there was no *Igsf21* expression in the anterior thalamic pole (AM; Fig. 4a, a′, j, j′) and a reduced cell expression density in dorsal, lateral and posterior thalamic nuclei. Scattered *Igsf21* + cells extended ventrally into the dorsal nuclei domain and intermixed with some remaining *Btbd3*-expressing cells (Fig. 4b, c, b′, c′, k, l, k′, l′). *Pde10a* gene expression was still upregulated in the VM and extended dorsally in Fgf8^null/neo^ mice, and decreased expression was observed in the VPM/L (*n* = 4; Fig. 4d–f′, k, l, k′, l′), whereas *Btbd3* was downregulated in the VPM/L and DL in the mutant mouse compared to the control (*n* = 4; Fig. 4g–i′, k–l, k′–l′). Overlay compositions of *Igsf21, Pde10a* and *Btbd3* expressions, generated using consecutive sections of different gene series from the same animal, to preserve the highest possible accuracy, showed that the molecular boundary between the dorsal and ventral thalamic nuclei was still detectable in mutant brains in the ventro-medial region, but was completely disrupted in the lateral regions, both at E15.5 and E17.5 (Fig. 3o, p and Fig. 4k′, l′).

These results indicate that Fgf8^null/neo^ embryos present strong alterations in TML regionalization, showing a distorted expression pattern for the *Igsf21, Pde10a* and *Btbd3* genes in the TML, revealing the absence of ventral and dorsal nuclear specification and the formation of the internal medullary lamina between these when *Fgf8* expression is reduced (Figs. 3, 4).

### Altered telencephalic expression of *Igsf21* and *Pde10a* in Fgf8^null/neo^ mice

The expression of *Igsf21, Pde10a* and *Btbd3* in control (*n* = 4) and mutant (*n* = 4) embryos at E17.5 was analyzed in transversal sections of the anterior telencephalon (Fig. 4). In control embryos, *Igsf21* was strongly expressed in the subplate (SP; arrowhead Fig. 4j, j″) and coexpressed with *Pde10a* in the cortical plate, while in the mutant embryos, *Igsf21* expression presented as a thick layer of neurons below and segregated from the cortical plate, labelled by *Pde10a* expression (Fig. 4a, a′, d, d’ and arrowhead in j, j′). No significant differences were observed in the expression pattern of *Btbd3* in the Cx (Fig. 4g–i′). Nevertheless, in mutant mice, the expression of *Pde10a* was strongly reduced in the dorsal layers of the medial Cx (Fig. 4d–f′). In addition, in the basal ganglia, while *Pde10a* was strongly expressed in the embryonic striatum of controls (St; Fig. 4d, j), in Fgf8^null/neo^ mice this expression was not detected (Fig. 4d′, j′).

### Reduced cell proliferation and increased cell death in the Fgf8^null/neo^ mouse thalamus

**Fig. 5:**
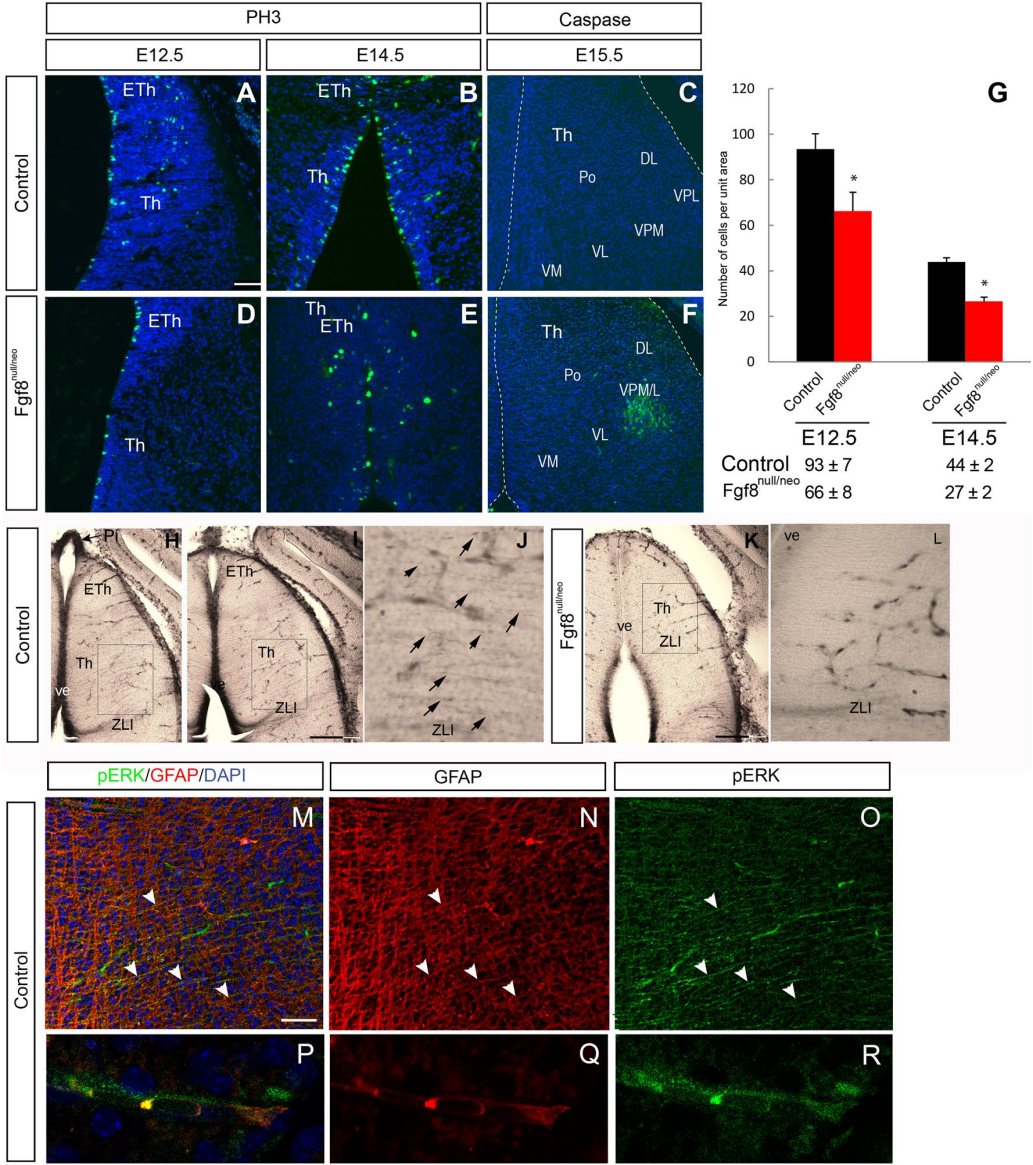
Proliferation and apoptosis were altered in the diencephalon of Fgf8^null/neo^ mutant embryos (**a, b, d, e**). Immunohistochemistry using an anti-PH3 antibody on transversal sections of the Di revealed severely reduced PH3 immunoreactivity in Fgf8 mutant embryos at E12.5 (**a, d**) and at E14.5 (**b, e**). Apoptotic cells were detected by caspase-3 immunofluorescence only in the Fgf8^null/neo^ lateral and ventral region of the thalamic mantle layer at E15.5 (**f**), but not in wt (**c**). Histograms presenting the average number (± SEM) of proliferating cells per unit area, * indicates values that were statistically different (*p* < 0.05) (**g**). pERK expression in E14.5 control Th, where ventricular epithelium (H and I) and fibers (**j**) appear immunopositive, as well as blood vessel cells. In Fgf8^null/neo^ mice, the expression of pERK in thalamic fibers is not detected, while vascular expression is remaining (**k, l**). Strong pERK expression in Pi was used as a positive control for pERK immunolabelling (arrow in **h**). Double immunohistochemistry (**m**) against GFAP (red; **n, q**) and pERK (green; **o, r**). High-powered image shows double labelling of GFAP and pERK in thalamic radial glia fibers (**p**). Scale bar: 200 µm in **h, i** and **k**; 50 µm in **a**–**f, j, l, m**–**o** and 15 µm in P–R

Previous studies have shown that *Fgf8* works as a cell proliferation signal (Lee et al. 1997; Xu et al. 2000; Sun et al. 2002; Martinez-Ferre and Martinez 2009) and as a cell survival factor (Sun et al. 2002; Storm et al. 2003; Chi et al. 2003). To explore cell proliferation in TLM, *Fgf8* mutants were analyzed at E12.5 and E14.5 by detecting the expression of the M-phase cell cycle marker, PH3. In control embryos, the rate of cell proliferation in the Di was higher at E12.5 (93 ± 7) than in later developmental stages (E14.5, 44 ± 2; Fig. 5a, b, g). Moreover, at both E12.5 and E14.5, control embryos (*n* = 3 for each stage) showed a significantly higher mitotic index (number of positive cells per unit area) in Th and Eth than mutants (*n* = 3 for each stage; *p* = 0.021 and *p* < 0.05), respectively (Fig. 5a, b, d, e, g); but no differences in mitotic distribution were observed.

To explore if cell death could explain regional alteration in *Fgf8* hypomorphic mice, we analyzed the expression of caspase-3, which was not detected in the control TML (Fig. 5c). We observed dying cells in the *Fgf8* mutant embryos in the VPM/L of the TML at E15.5 (Fig. 5f) and E17.5 (Fig. 4l″).

These results indicate that both proliferation and cell death in the Th are affected by the decreased expression of *Fgf8*. Interestingly, cell death appeared selective in the ventrolateral TML, the most distant region from the ventricular source of *Fgf8* signal.

To evidence a reduction in the *Fgf8* signal in the TML, we used immunohistochemistry to study the expression of pERK, which is induced when the Fgf signal binds tyrosine kinase Fgf receptors (Echevarria et al. 2005). At E14.5, the dorsal Th ventricular epithelium was strongly positive for pERK in WT mice (*n* = 4; Fig. 5h, i). This expression was also observed with a fibrillar pattern in the neuroepithelium crossing the TML, towards the pial surface (Fig. 5j). That expression seemed to overlap with radial glia fibers and appeared more strongly stained at the level of the thalamic VPL/M and ZLI regions (Fig. 5h–j). The expression of pERK was completely absent in the Fgf8^null/neo^ mice dorsal Th (*n* = 4), both in the ventricular epithelium and TML fibers (Fig. 5k, l). To test whether radial glia fibers were expressing pERK, we performed double immunohistochemistry against pERK and GFAP, and observed that, as expected, there was co-expression of these antibodies (Fig. 5m–r). Moreover, since the expression of pERK in blood vessels has recently been reported by our group (Pombero et al. 2018), we decided to explore this in the thalamus, as a control for pERK expression in mutants. The vascular pERK expression was similar in WT and Fgf8^null/neo^ TML (Fig. 5h–l). The presence of normal radial glia cells and fibers in Fgf8^null/neo^ mice at these stages were detected through anti-GFAP and vimentin immunohistochemistry (data not shown). These results suggest that *Fgf8* morphogenetic activity in the ventricular epithelium is translated to the TML by the expression of pERK in radial glia fibers, which seems to be required for cell survival and appropriate nuclear patterning.

**Fig. 6:**
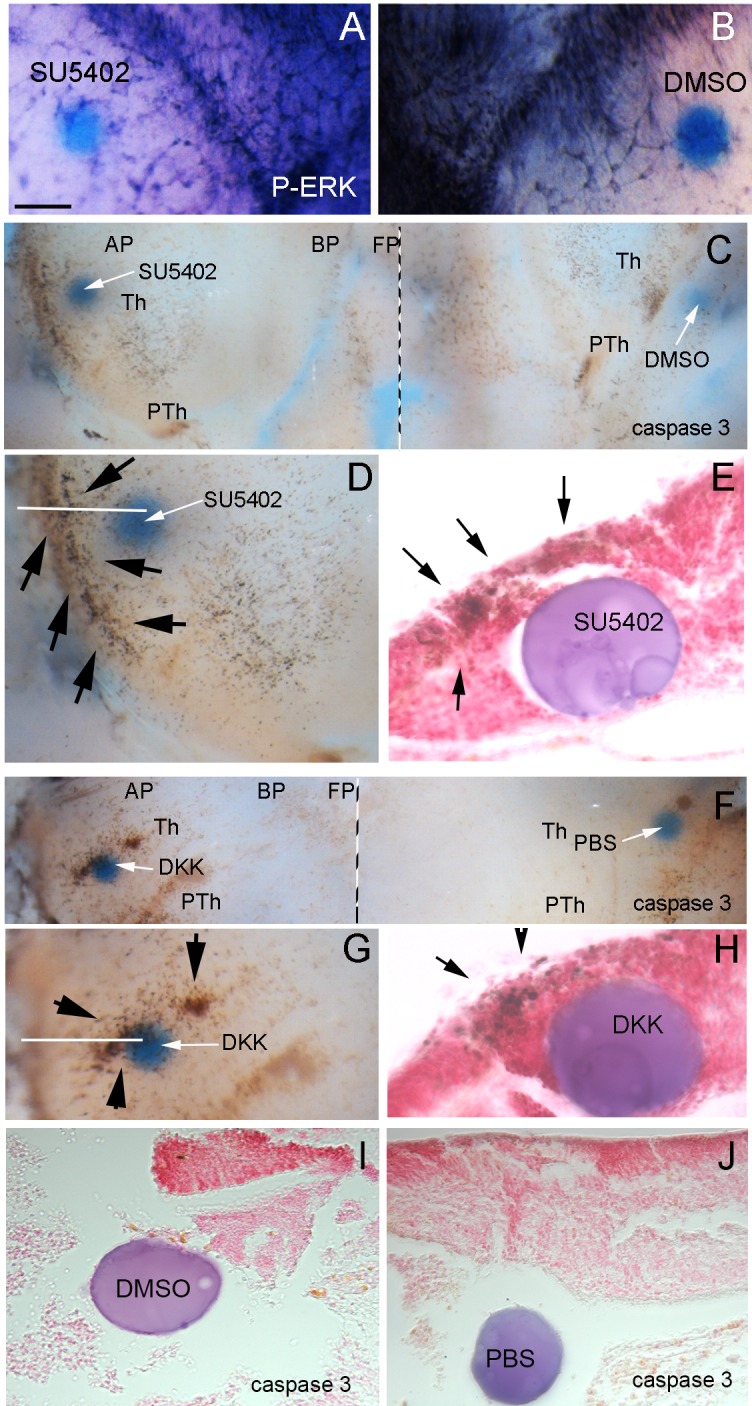
*Fgf8* and *Wnt* signal loss of function in neuroepithelial mouse explants. **a, b** The anterior Th and ZLI radial glia cells and fibers strongly express pERK on the control side of the neuroepithelial explants (right side where DMSO control microbead was implanted); after blocking Fgf receptors with a SU5402 bead implant (left side), pERK expression in the thalamic and ZLI radial glia decreases, while radial glia fibers are not detectable in the thalamic mantle layer and the vascular expression remains. **c, e** After Fgf signal blocking by SU5402 embedded microbeads in the Th, strong increases in caspase-3-expressing cells are detectable around the experimental bead (left side in **c, d**) in comparison with the control side (right side in **c**). After sectioning and neutral red staining of the bead and nearby thalamic wall, caspase-3-expressing cells can be seen in both ventricular epithelium and mantle layer cells (**e**; black arrows in **d, e**). **f, h** After Wnt signal blocking by DKK embedded microbeads in the Th, a strong increase in caspase-3-expressing cells is detectable around the experimental bead (left side in **f, g**) in comparison with the control side (right side in **f**). After sectioning and neutral red staining of the bead and nearby thalamic wall, caspase-3-expressing cells can be seen in both ventricular epithelium and mantle layer cells (arrows) Control beads (**i, j**) did not induce caspase 3 expression. **i** Corresponds to the control bead for SU5402 experiments (embedded in DMSO) and **j** to the DKK experiments (embedded in PBS). Scale bar 200 µm in **c, f**, 170 µm in **a, b, d, e**; 100 µm in **i, j**; 50 µm in **e, h**

To demonstrate that the *Fgf8* signal regulates pERK expression in the thalamic radial glia, we performed “open book” E14.5 neuroepithelial explants of the WT (Fig. 6); the right side was the control side and the left side was the experimental side. Due to the variability and the complexity of the phenotype in mutant embryos, we decided to conduct our experimental approach in WT embryos in which anatomical landmarks were consistently identified. This design allowed us to recapitulate the effect of Fgf8 or Wnt3 inhibition that is expressed downstream of Fgf8 (Martinez-Ferre and Martinez 2009). We implanted microbeads embedded in SU5402 to block FGF8 or DKK to block WNT signaling. One day after implanting microbeads embedded in *Fgf8* signal blocker (SU5402; *n* = 5), the pERK expression was strongly reduced in the thalamic and ZLI radial gland near the bead (Fig. 6a) compared to the control side, where the beads embedded with DMSO were implanted (Fig. 6b). This indicates that a *Fgf8* signal is required to maintain pERK expression in radial glia.

Next, to explore potential mechanisms for understanding TML cell death after pERK reduction, we analyzed caspase 3 immunohistochemistry after SU5402 microbead implantation. Cell death was more abundant in the experimental Th (*n* = 5; Fig. 6c, d, e), close to the SU5402 beads (arrows in Fig. 6d, e), than on the control side (*n* = 5;Fig. 5c, i), confirming that FGF8/pERK signal is required for cell survival in the developing Th. Interestingly, *Fgf8* activity in the dorsal Th induces *Wnt3* expression, which was not seen in Fgf8^null/neo^ mutant mice (Martinez-Ferre and Martinez 2009); for this reason, we explored the effects of Wnt signaling inhibition by DKK (Wnt antagonist) microbead (*n* = 6) implants. Increased cell death was induced on the experimental side around the DKK bead (Fig. 6f–h, arrows in G,H), something that was not detected with control implants of PBS-soaked beads (Fig. 6j).

### Altered thalamocortical axons in Fgf8^null/neo^ mice

**Fig. 7:**
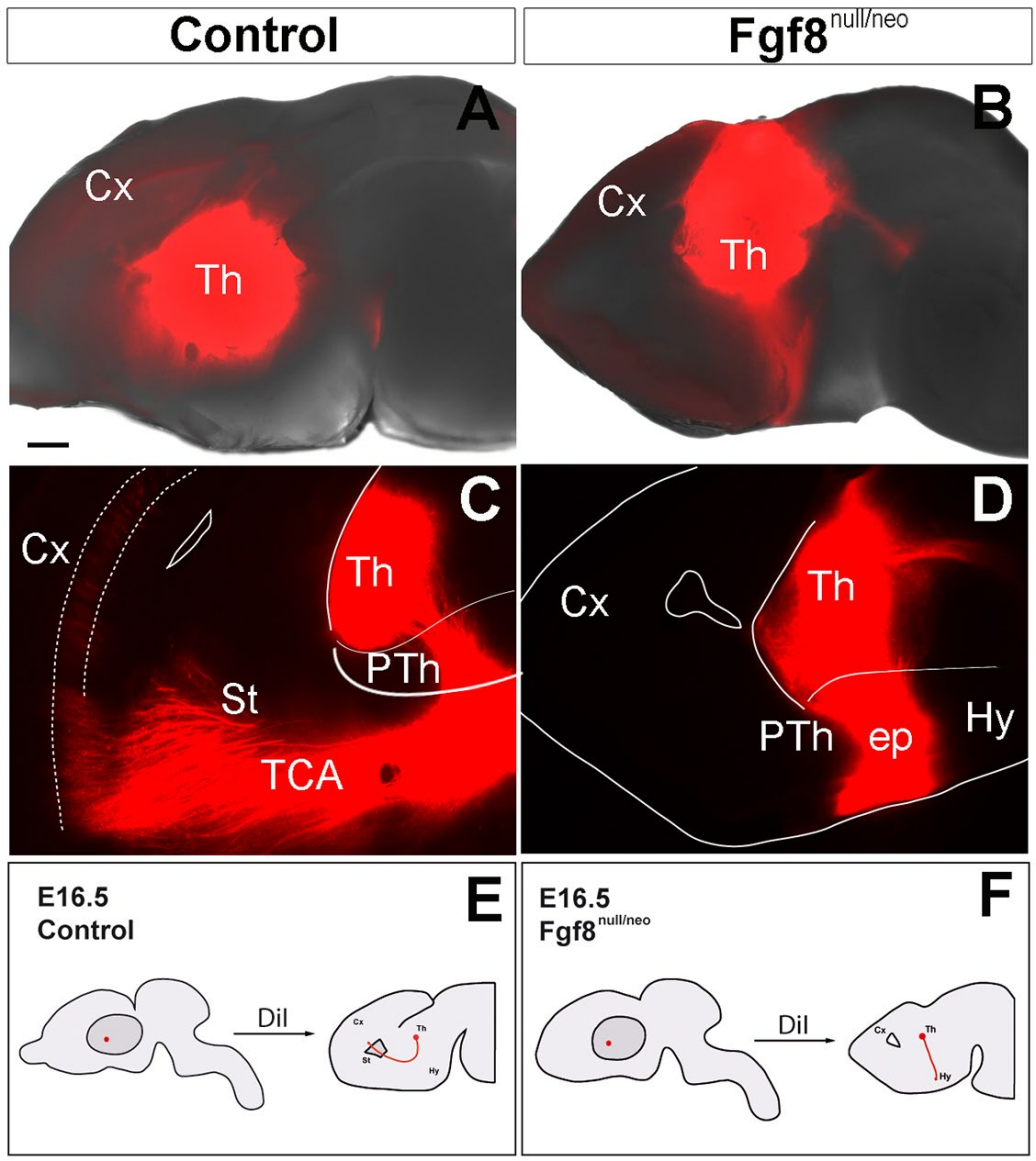
Abnormal pathfinding of TCAs in Fgf8^null/neo^ embryos. **a, b** Medial views of the brain hemispheres with DiI crystals in the Th of control (**a**) and Fgf8^null/neo^ (**b**) mice brains. **c, d** Sagittal sections at E16.5 of the corresponding brains after DiI diffusion. **e, f** Experimental paradigm used to test TCAs in control and Fgf8^null/neo^ E16.5 embryos; the axonal tract is represented in red. Scale bar: 200 µm in **a, b**; 150 µm in** c** and** d**

The TC is also a marker for exploring thalamic regionalization, since mutations in thalamic genes (*Gbx2* and *Shh*) significantly alter this tract (Miyashita-Lin et al. 1999; Szabo et al. 2009). Although (Garel et al. 2003) reported that TC was normal in mildly hypomorphic Fgf8^neo/neo^ mice, it is unknown what effect a stronger reduction in the *Fgf8* signal would have in the TC of Fgf8^null/neo^ mice. In fact, FGF signals promote the presence of specific guidance cues critical for normal optic tract development (Atkinson-Leadbeater et al. 2010). To explore the potential role of *Fgf8* reduction in TC development, DiI crystals were implanted into the Th of E16.5 control and mutant mice (Fig. 7). The control TC followed rostrally from the Th to the lateral PTh, peduncular hypothalamus (PHy) and turned dorsally and laterally to enter the subpallium and Cx along the internal capsule (*n* = 8, Fig. 7a, c, e). All the controls (*n* = 8) examined showed similar projection patterns. In contrast, DiI injections in the Fgf8^null/neo^ embryos resulted in the derailment of thalamic axons in mutant mice (14/20). These axons followed rostrally towards the anterior forebrain through the PHy, but instead of making a dorsal turn, they continued on an ectopic rostral projection (ep) towards the anterior hypothalamus, into the preoptic area (POA; *n* = 14,Fig. 7b, d, f). In 30% of cases (*n* = 6), some TC axons did turn dorsally and enter the pallium.

### Altered Ntn1 and DCC expression in the thalamocortical pathway of Fgf8^null/neo^ mutant mice

**Fig. 8:**
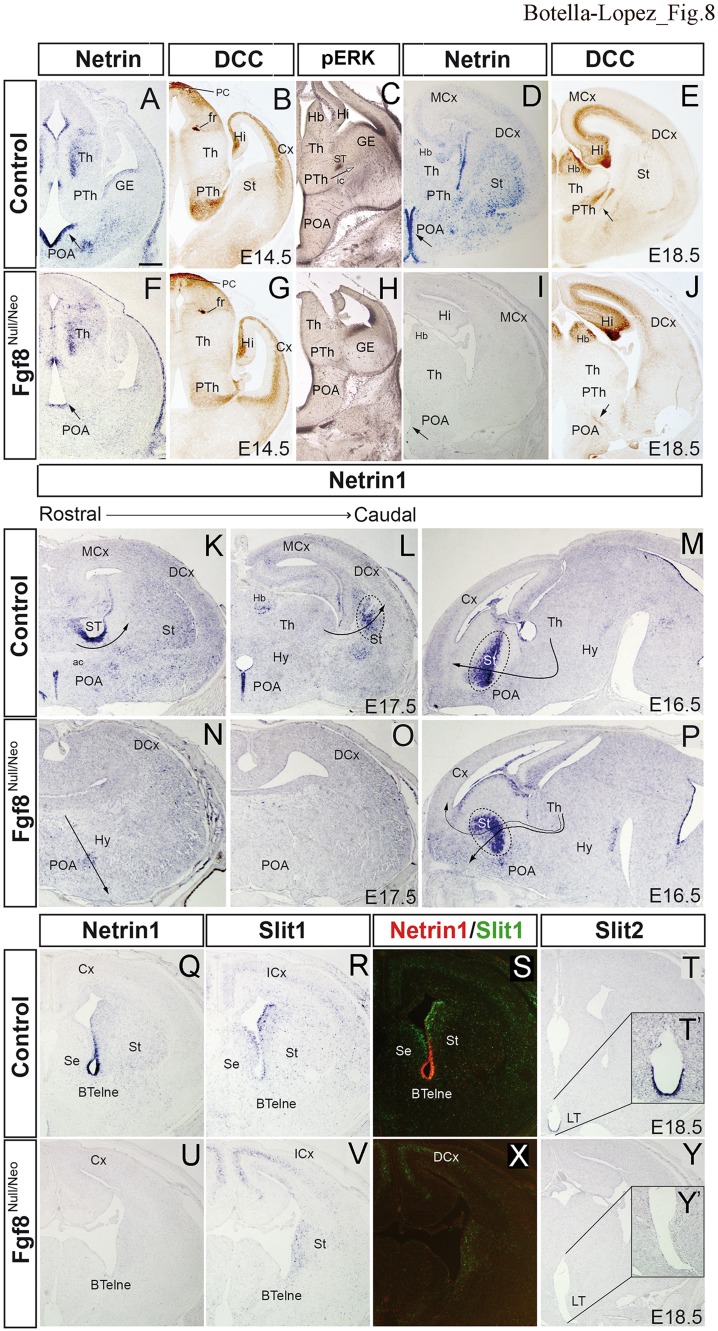
Altered guidance molecules in Fgf8^null/neo^ mice. *Ntn1* (**a, d, f, i**), DCC (**b, e, g, j**) and pERK (**c, h**) immunostaining in transversal sections of control and Fgf8^null/neo^ mice at E14.5 and E18.5. The arrows indicate different expression levels of *Ntn1* in the POA and Hy (**a, d**). At E18.5, DCC + TCAs entered the Cx (arrow in **e**); in Fgf8 mutant DCC + TCAs followed an aberrant path towards the Hy (arrow in **j**). pERk expression in the radial glia and fibers is strong in *Ntn1* positive epithelia (GE and POA), as well as in the stria terminalis (ST) and peduncular area, where thalamo-cortical fibers enter the subpallium (arrow). In the mutant, only POA and GE show pERK expression. *Ntn11* expression in transverse (**k, l, n, o**) and sagittal sections (**m, p**) of control and Fgf8^null/neo^ mice at E17.5 and E16.5. Arrows indicate the likely direction of the TCAs, following the attracting effect of *Ntn1*. Dashed lines delimit the regions with strong *Ntn1* labelling. *Ntn1* expression (**q, u**), Slit1 expression (**r, v**) and color-coded overlays of hybridizations for *Ntn1* (red) and *Slit1* (green) (**s, x**) in transversal sections of E18.5 control and hypomorph brains. *Slit2* expression (**t, y**) in E18.5 transversal sections of control and mutant mice. Higher magnification view of boxed area in the control and Fgf8^null/neo^ embryos (**t**′,** y**′). Scale bar: 200 µm

The aberrant projection route of TC detected in the Fgf8^null/neo^ mutant could be due either to a misspecification of projecting thalamic neurons or alterations in the axonal navigation mechanisms. Since thalamic neurons normally send axons through the anterior thalamic peduncle (Fig. 7), the aberrant TC may not be due to a primary neuronal phenotype. Several studies have demonstrated that Netrin 1 (Ntn1) /DCC and Slit 1, 2/Robo1, 2 are important signaling molecules involved in the guidance of thalamocortical axons during embryonic development [reviewed in (Leyva-Díaz and López-Bendito 2013; Leyva-Díaz et al. 2014)]. The gradient expression of Ntn1 in VTel attracts the axons expressing DCC receptors (Powell et al. 2008). For this reason, we have analyzed the expression pattern of long-range guidance signals, the *Ntn1* gene and its receptor (DCC). At E14.5 in the Di and anterior prosencephalon, *Ntn1* expression was detected in the periventricular TML, with a gradient decreasing towards the lateral surface, both in control (Fig. 8a, *n* = 4) and mutant animals (Fig. 8f, *n* = 4). This may be the signal that induces axonal growth of the thalamic peduncle. However, the expression of *Ntn1* in the POA and alar hypothalamic ventricular epithelium was downregulated in Fgf8^null/neo^ mutants (arrow in Fig. 8a, f). This reduction in the POA was more pronounced at E18.5 (arrow in Fig. 8d, i; *n* = 3 control and 3 mutants). DCC was detected in the PTh (Fig. 8b, *n* = 3), with less intensity in the mutant mouse (Fig. 8g, *n* = 4). At E18.5, DCC-positive axons were identified in the internal capsule (arrow in Fig. 8e, *n* = 3); however, in the Fgf8^null/neo^ mouse, DCC-positive axons followed rostrally along an aberrant pathway into the lateral POA (arrow in Fig. 8j, *n* = 4). In the control telencephalon, *Ntn1* was also detected in the ventricular epithelium and mantle layer of the bed nucleus of stria terminalis (ST) and the pallidum (Fig. 7i), as well as in the caudal part of the St (Fig. 8i, j). In the mutant brain at E14.5, E16.5, and E18.5, *Ntn1* expression was not detected in any of these regions, except in disperse POA cells (Fig. 8l). Next, we analyzed its expression in three mutant mice with a mild phenotype, where part of the TC turned to the Cx (30%; 6/20) and part followed the aberrant pathway to the anterior prosencephalic surface. In these mutants, at E16.5, *Ntn1* expression was strongly detected in the peduncular domain of St, in both control (Fig. 8m) and mutant (Fig. 8p) mice. However, the decreasing pallidum-to-striatum gradient observed in WT (Fig. 8m) was misdirected in the mutants, decreasing from the ventricular to a superficial region (Fig. 8p). Then, since the TC axons followed the decreasing *Ntn1* gradient in the telencephalic peduncle, they were directed towards the pallium in WT and the anterior prosencephalon-POA in the mutant brain. This means that a reduced *Fgf8* signal is involved in the absence or reduction of *Ntn1* expression in the telencephalic peduncular region and, therefore, in the alteration in the local TC guides. This control of local signal expression by *Fgf8* signaling may be also driven by pERK expression in radial glia, since strong pERK expression was detected in these regions (Fig. 8c), whereas it was not detected in Fgf8^null/neo^ mice (Fig. 8h).

### The co-expression of Netrin 1/Slit 1 was impaired in Fgf8^null/neo^ mice

The expression of the *Slits*/*Slits* receptor family (*Slit1, Slit2, Robo1* and *Robo2*) was also analyzed. These signaling molecules repel the axons expressing *Robo1*/*Robo2* when they interact with *Slit1*/*Slit2*, deviating them from the hypothalamus and midline (Braisted et al. 2009; Leyva-Díaz and López-Bendito 2013). At E18.5, the control mice expressed *Ntn1* in the neuroepithelial zone of the anterior basal telencephalon (BTel) (Fig. 8q, s); however, no signal was detected in Fgf8^null/neo^ mutants (Fig. 8u, x). While *Slit1* was detected in the SVZ of the St and Septum (Se; Fig. 8r, s) in controls, this gene was only detected in the St of the mutants (Fig. 8v, x). A combined analysis of *Ntn*1 and *Slit1* expression in a color-coded overlay revealed the complementary expression of these genes in the control BTel (Fig. 8S), but not in the mutant mouse (Fig. 8x). *Slit2* expression was detected in the lamina terminalis (LT; Fig. 8t, t′) in control mice, while it was absent in mutants (Fig. 8y, y′). No differences in the expression patterns of *Robo1* and *Robo2* were detected between the control and Fgf8^null/neo^ embryos (data not shown).

## Discussion

### Role of *Fgf8* in thalamic regionalization

**Fig. 9:**
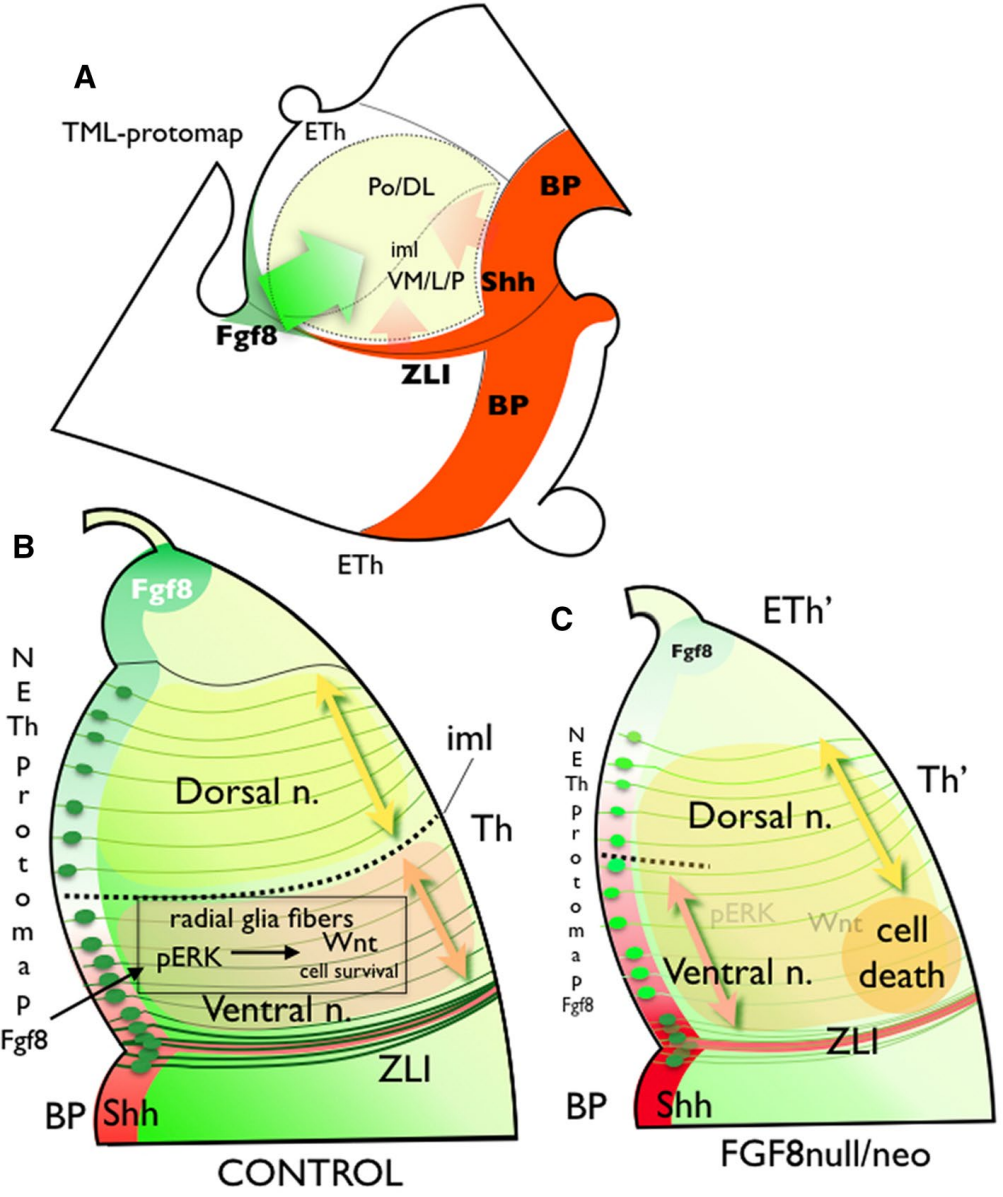
Schematic representation of the TML protomap in the Fgf8 hypomorph phenotype. **a** Schematic representation of the TML protomap in a lateral view of E14.5 Di. The internal medullary lamina (iml) separates the dorsal nuclear complex (PoDL) from the ventral nuclear complex (VM/LP). Colored arrows represent the *Fgf8* gradient signal (green arrow), with the *Shh* gradient signal (red) acting over TML. **b***Fgf8* antero/dorso-postero/ventral gradient, together with a *Shh* complementary gradient, establishes the neuroepithelial and mantle layer molecular maps. *Fgf8* signal acts through a ventricular and neuroepithelial diffusion that activates pERK expression in radial glia cells and fibers, allowing the dorsal and ventral nuclei specification and separation by the iml. pERK upregulates *Wnt3* expression, which represents the cell survival signal. **c** The reduction of *Fgf8* signaling modifies morphogenetic information and produces a strong reduction of pERK expression in the radial glia with the subsequent absence of *Wnt3* activation. This represents cell death in the lateral and ventral Th, as well as abnormalities in thalamic regionalization (Eth′ and Th′), increasing the area occupied by ventral nuclei in the periventricular stratum; and the ventral induction of dorsal markers in intermediate and superficial strata

Thalamic regionalization is controlled through the molecular specification of progenitors in the p2 alar plate. The expression of specific cell fate genes is regulated through the distribution of diffusible morphogenetic signals at the ventricular epithelium: FGF8, SHH, WNT and BMP, encoding positional information (Martínez and Puelles 2000; Echevarría et al. 2003; Scholpp et al. 2006; Szabo et al. 2009; Vue et al. 2009; Scholpp and Lumsden 2010; Nakagawa and Shimogori 2012; Bluske et al. 2012; Puelles and Martinez 2013; Martinez-Ferre et al. 2013). This positional information might be translated to the TML, where neurons populate specific nuclear masses. The TML protomap determines the molecular signature that governs hodological differentiation and the function of thalamic neurons. Neurons in the VM/L, receiving primary sensory lemniscal pathways, express the *Btbd3* gene, differentiate into c-type neurons and project to layer III and IV of the primary sensorial Cx (Clascá et al. 2012). These regions showed a strong pERK signal requirement for their survival and nuclear segregation (Fig. 9). In the Eurexpress database, the expression of *Wnt3* and pERK is stronger in the ventral and lateral TML than in the dorsal TML at E14.5 (Supplementary Fig. 1, insert), suggesting that pERK may locally increase the Wnt signal. Conversely, ventro-medial and anterior thalamic nuclei are less sensitive to pERK/WNT3 signals and require other factors as SHH and GBX2 (Szabo et al. 2009; Vue et al. 2009; Nakagawa and Shimogori 2012). Neurons in these nuclei receive cerebellar and striatal afferents, and project, as m-type (multiareal) thalamic cells, to layers 5 and 1 of the frontal motor Cx (Clascá et al. 2012). Neurons in the dorsal and posterior nuclei express *Igsf21* differentiate into m-type and il-type neurons and project to superficial and deep cortical layers of medial and caudal association areas (Clascá et al. 2012). In this study, we have demonstrated that neurons in the dorsal and posterior nuclei survive the reduced *Fgf8* signal and extend rostrally due to re-specification of anterior domains (Fig. 9). As a consequence of inadequate neuronal segregation into nuclear masses, the molecular intrathalamic boundary that differentiates dorsal from ventral nuclei, the thalamic internal medullary lamina did not develop properly.

The selected genes: *Igsf21, Pde10* and *Btbd3*, served as markers to reveal how the neuroepithelial positional map was propagated to postmitotic neurons of the mantle layer, as postulated by CD15 expression in the human Th (Forutan et al. 2001) and *Eomes* expression in the Cx (Elsen et al. 2013). In the Th, as previously described in cortical layering (Rakic 2009), the radial glia fibers may represent a scaffold on which to organize the structural complexity of TML development. Through a cell-autonomous signal, radial fibers enable the translation of 2D positional information from the ventricular epithelium to a 3D structure in the thalamic nuclear complex. The expression of pERK along the radial fibers may be a representation of the *Fgf8* signaling code, which from the dorsal-anterior thalamic midline and the dorsal ZLI produces different pERK activation levels in ventricular radial glial cells. Although it cannot be assured that FGF8 protein acts in a direct manner on the TML, the reduction of *Fgf8* expression in diencephalic dorsal midline is bound to a decrease of pERK in TML and to an altered thalamic development. FGF signaling is mediated via tyrosine kinase receptors (FGFRs) that act through a number of transduction pathways, like the highly conserved Ras-ERK mitogen-activated protein kinase (MAPK) (reviewed by Niehrs and Meinhardt 2002; Echevarria et al. 2005). Moreover, the absence of *Fgf15* signal did not show this phenotype (Martinez-Ferre et al. 2016) suggesting that *Fgf8* is the direct or indirect signal to maintain pERK expression in radial glia fibers.

Since *Wnt3* expression in the lateral TLM was stronger in the ventral than dorsal nuclear territory (insert in Fig.S2), this may be related to the local specification and survival of ventro-lateral thalamic cells. We have shown that *Wnt3* expression is induced by *Fgf8* activity in thalamic neuroepithelium (Martinez-Ferre and Martinez 2012). Hence, the FGF8/pERK/WNT signal may be necessary for cell survival. This is in agreement with (Guo et al. 2017), who reported that pERK activates the *Wnt* canonical pathway in cancer cells. Moreover, (Libro et al. 2016) showed that *Wnt-βcatenin* signal perturbations are associated with cell death in the CNS. Therefore, pERK expression in the radial glia fibers is required to maintain the *Wnt* signal in TML, which is necessary for the survival of VL Th nuclei.

Gezelius et al. (2016) also demonstrated molecular heterogeneity in the thalamic sensory nuclei at two developmental stages: E14.5 and E18.5. In their screening, they did not detect expression of the three genes we have identified, probably because their analysis method did not generate sufficient transcriptomic differences along expression gradients in reduced volumes.

While no reports analyze the expression of *Igsf21* in the brain, *Pde10* is a cyclic nucleotide phosphodiesterase (PDE) that determines cyclic nucleotide signaling. PDE signaling regulates cAMP and cGMP concentrations in thalamic neurons *in vitro* (Hepp et al. 2007), a signaling pathway involved in neuronal plasticity and axonal growth (Mango et al. 2014). More interestingly, *Btbd3* expression is involved in the control of dendrite orientation in the mammalian neocortex (Matsui et al. 2013). In fact, *Btbd3* expression is enhanced in neuronal regions where functional-related remodeling of connections is required. Thus, the expression of *Btbd3* in the Th may be required to develop topological sensorial maps in VL and dorsal geniculate nuclei. We propose that the reduction of *Fgf8* signal is responsible for the thalamic disorganization and, as a consequence, *Igsf21, Pde10a, Btbd3* showed an altered expression. The demonstrated role of these genes in some developmental processes indicates that more studies are needed to reveal their influence in the regionalization of the thalamus.

*Fgf8* expression is involved in the organization of anterior thalamic and prethalamic nuclei (Kataoka and Shimogori 2008), as well as the ETh (Martinez-Ferre and Martinez 2009). Although our previous studies showed that reduced *Fgf8* signaling in the Di does not lead to an increase in cell death in early developmental stages [E11.5-13.5; (Martinez-Ferre and Martinez 2012)], as it occurs in the midbrain/hindbrain region (Chi et al. 2003), here we detected positive caspase signaling in the ventro-lateral thalamic mantle layer at later stages (E15.5; Fig. 5c, f). Therefore, *Fgf8* signaling is required for the survival of thalamic neurons at E15.5 and E17.5, in a dose-dependent manner. Since the thalamic phenotype was detected in regions without cell death marker expression, and cell migration alterations were not observed in our mutants, patterning anomalies in Fgf8^null/neo^ may be a consequence of re-specification (extension of medial and dorsal nuclei) and neuronal death in VL Th.

Our results, therefore, indicate that *Fgf8* signal levels affect thalamic regionalization, acting on TML neurons. That molecular signaling contribute to the increase of TML complexity by projecting the 2D ventricular patterning onto a 3D organized structure following differences in signal intensity in radial glia fibers. This 3D pattern is exclusively coded by *Fgf8* expression, since although *Fgf15* is expressed in the TML, and regulated by *Shh* (Gimeno and Martinez 2007), there was no nuclear disorganization in the thalamus of *Fgf15-*/*-* mice, and it is not reproduced in Fgf8^null/neo^ mice (Martinez-Ferre et al. 2016).

### Thalamocortical Circuit Abnormalities in Fgf8 hypomorphic mice

In the Fgf8^null/neo^ mutant, the axons leave the Th, cross the PTh and continue to the pPHT, rather than turn to enter the ventral telencephalon and enter the Cx (Braisted et al. 1999; Tuttle et al. 1999). However, only a 30% of Fgf8^null/neo^ mice (6/20) did present partial distribution of the thalamic axons towards the Cx. Garel et al. (2003) reported a normal TC projection in hypomorphic Fgf8^neo/neo^ mice. These differences may be explained by the level of Fgf8 reduction, which is 40% of normal levels in Fgf8^neo/neo^ and 20% in Fgf8^null/neo^ (Meyers et al. 1998; Garel et al. 2003).

TC abnormalities, or functional connectivity impairments, have recently been related to various human diseases, such as idiopathic epilepsy (O’Muircheartaigh et al. 2012; Kim et al. 2014), cognitive dysfunction in anorexia (Biezonski et al. 2016), a high risk of psychosis (Anticevic et al. 2015) and autism spectrum disorder (Nair et al. 2013, 2015). Therefore, different degrees of alteration in FGF8 signaling may be involved in the developmental processes underlying the multifactorial mechanisms of these diseases.

### Adhesion molecule patterns were altered in the TC pathway of Fgf8^null/neo^ mice

In Fgf8^null/neo^ mice, thalamic axons seemed incapable of identifying the pathway towards the Cx and subsequently migrated in the wrong direction. The TC pathway crosses distinct territories and requires precisely organized attraction/repulsion signals (Dickson 2002; Molnár et al. 2012; Garel and López-Bendito 2014).

We analyzed the expression of Netrins and Slits and their neuronal receptors (DCC and Robo, respectively) in Fgf8^null/neo^ mice. Several studies have shown that *Ntn1* acts as an intermediate target for thalamic axons (Bonnin et al. 2007; Braisted et al. 2000; Culotti and Merz 1998). The long-range gradient of *Ntn1* in the subpallium is required for the topographic sorting of axons to distinct cortical domains (Powell et al. 2008). In the Fgf8^null/neo^ mutant, we demonstrated reduced of *Ntn1* expression and the disappearance of its gradient in the St. These changes in *Ntn1* expression, associated with the lack of radial glia pERK expression, could negatively influence the progression of the thalamo-cortical axons, as previously described by Powell et al. (2008). Congruent with the significance of signal distribution along the radial glia fibers, it has been proven that the *Ntn1* signal in radial glia fibers at the pial surface guides commissural axons to the ventral midline in the hindbrain (Dominici et al., 2017). Our results strongly suggest that *Ntn1* expression involved in the dorsal growth of TC to enter into the pallium requires normal pERK expression in the radial glia fibers. Previous work (Stein et al. 2001; Bonnin et al. 2007; Powell et al. 2008) reported that DCC is required to guide rostral thalamic axons to the *Ntn1*-rich rostral domain of the subpallium. The reduction of DCC expression in Fgf8 hypomorphic mice contributes to the aberrant migration route of the thalamo-cortical axons in Fgf8^null/neo^ embryos.

Another molecule analyzed in these mutants was Slit (Bielle et al. 2011). *Slit1* is highly expressed in the ganglionic eminence (GE) at the stages in which TC fibers are elongating, probably repelling these fibers from the proliferative region (Bagri et al. 2002). Additionally, it has been shown that *Slit2* repels thalamic axons in the hypothalamic area, preventing them from invading the hypothalamus (Braisted et al. 1999, 2009; Bagri et al. 2002; López-Bendito et al. 2007). Therefore, the loss of *Slit2* expression in the LT of hypomorphic mice allows thalamic axons to invade the anterior hypothalamic region and cross the midline.

*Fgf8* signal is involved in the specification of the neural territories where axonal guidance molecules are expressed. Therefore, since receptors of the analyzed molecules were expressed in the axons, it is suggested that the thalamocortical axons themselves develop correctly and they fail in reaching their pathway because of the misexpression of signaling molecules in the intermediate targets such as *Ntn1* or *Slits*.

In this study, we did not investigate cortico-thalamic axons, which may also be altered in Fgf8 hypomorphic mice; for this reason, we do not know what role these may play in this abnormal projection in the TC anomalies detected (Molnár et al. 1998).

## Electronic supplementary material

Below is the link to the electronic supplementary material.

Supplementary material 1 (TIF 8332 KB)

Supplementary material 2 (TIF 3642 KB)

## Abbreviations

AM: Anteromedial nucleus
AV: Anteroventral nucleus
BTel: Basal telencephalon neuroepithelium
Cx: Cortex
DCx: Dorsal cortex
Di: Diencephalon
DL: Dorsolateral nucleus
DPall: Dorsal pallium
DTel: Dorsal telencephalon
ETh: Epithalamus
Hb: Habenula
Hi: Hippocampus
Hy: Hypothalamus
lCx: Lateral cortex
iml: Internal medullary lamina
Inh: FGFR inhibitor
LT: Lamina terminalis
MCx: Medial cortex
MPall: Medial pallium
P1B-P3B: Basal plates of p1–p3
pc: Posterior commissure
PHy: Peduncular hypothalamus
Pi: Pineal gland
Po: Posterior nucleus
PT: Pretectum
PTh: Prethalamus
PVP: Posterior paraventricular nucleus
rf: Retroflexus fascicle
sm: Stria medullaris
Se: Septum
SePall: Pallial septum
St: Striatum
TC: Thalamocortical projection
TCh: Telencephalic choroidal tissue
TG: Tectal gray
Th: Thalamus
TML: Thalamic mantle layer
VCx: Ventral cortex
VM: Ventromedial nucleus
VL: Ventrolateral nucleus
VPL: Ventral posterolateral nucleus
VPM: Ventral posterior medial nucleus
VTel: Ventral telencephalon
ZLI: Zona limitans.
