# Supplementary figures and images for: Radial glia fibers translate Fgf8 morphogenetic signals to generate a thalamic nuclear complex protomap in the mantle layer
Source: Brain Struct Funct. 2018 Nov 23;224(2):661–79. doi: 10.1007/s00429-018-1794-y (PMC6420463; doi:10.1007/s00429-018-1794-y)

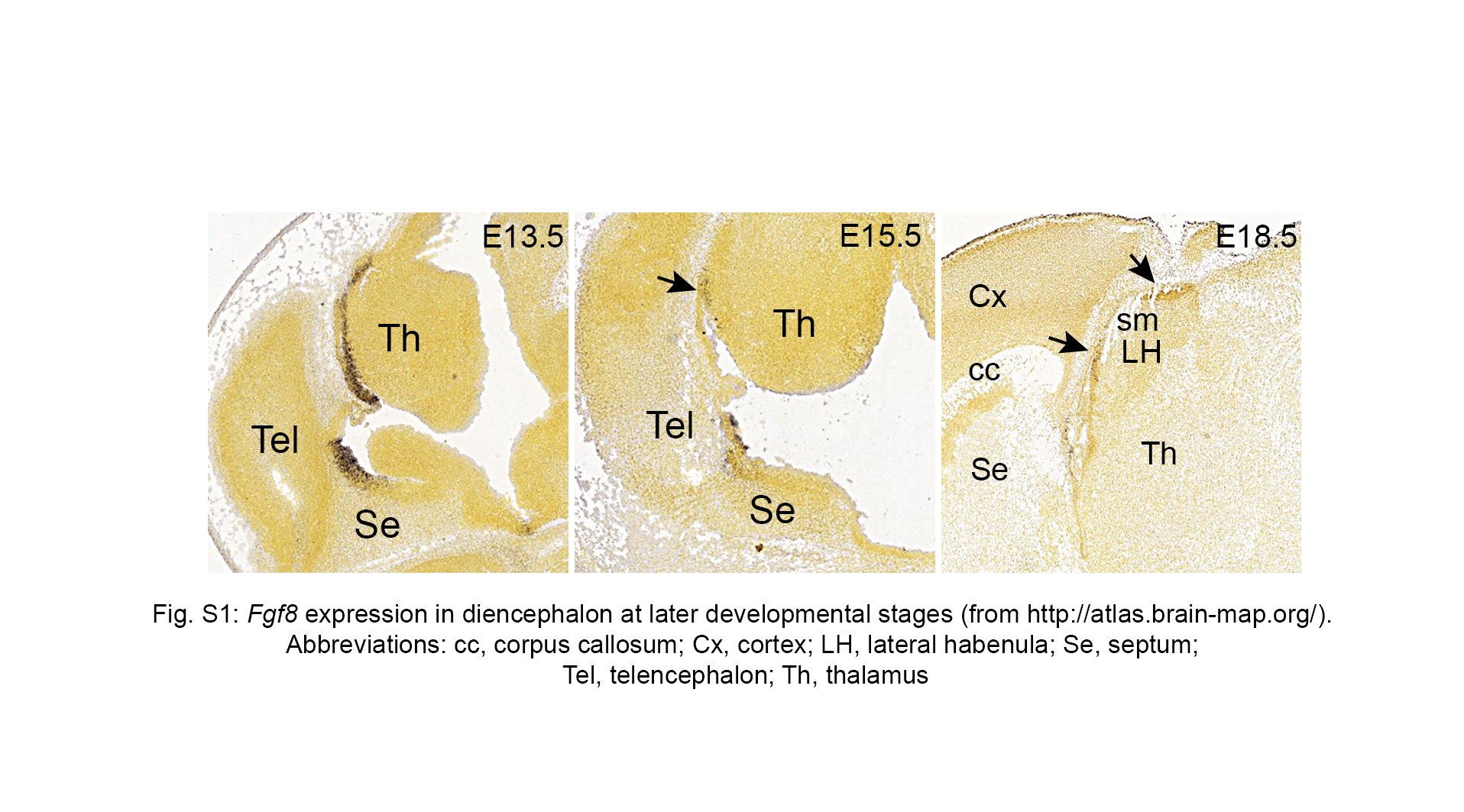

Supplement: Supplementary file 1 — Supplementary material 1 (TIF 8332 KB) [file 429_2018_1794_MOESM1_ESM.tif]

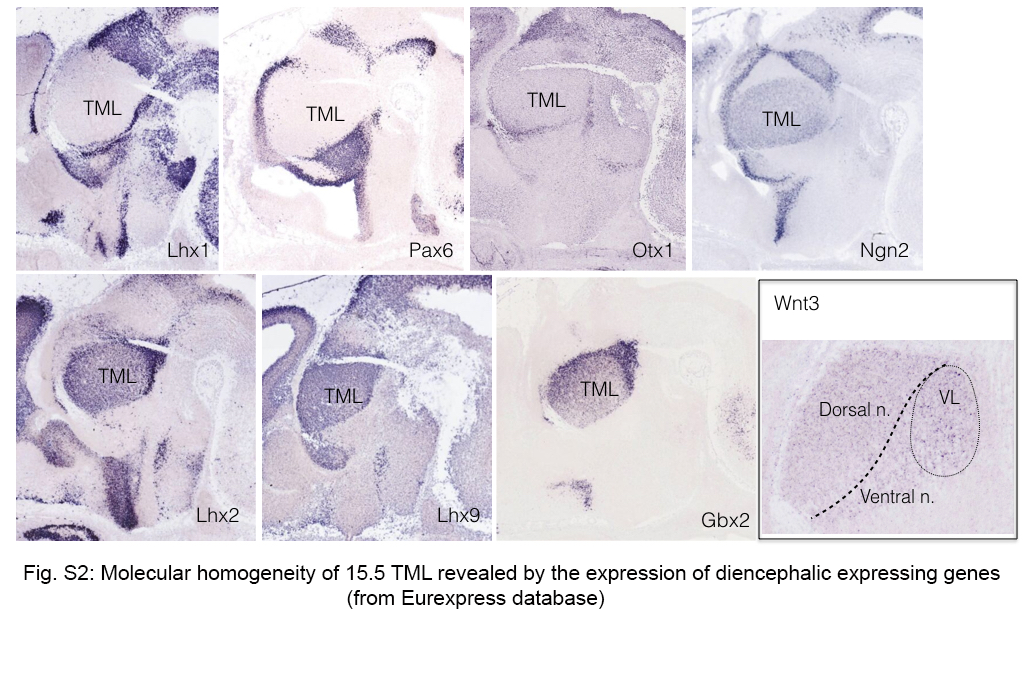

Supplement: Supplementary file 2 — Supplementary material 2 (TIF 3642 KB) [file 429_2018_1794_MOESM2_ESM.tif]
